# Electroacupuncture improves thermal and mechanical sensitivities in a rat model of postherpetic neuralgia

**DOI:** 10.1186/1744-8069-9-18

**Published:** 2013-04-03

**Authors:** Cai-hua Wu, Zheng-tao Lv, Yin Zhao, Yan Gao, Jia-qing Li, Fang Gao, Xian-fang Meng, Bo Tian, Jing Shi, Hui-lin Pan, Man Li

**Affiliations:** 1 Department of Neurobiology, School of Basic Medicine, Tongji Medical College of Huazhong University of Science and Technology, 13 Hangkong Road, Wuhan, 430030, P.R. China; 2 Department of Anesthesiology and Perioperative Medicine, The University of Texas MD Anderson Cancer Center, 1515 Holcombe Boulevard, Houston, TX77030, USA

**Keywords:** Neuropathic pain, Postherpetic neuralgia, Capsaicin, Acupuncture, Analgesia, Allodynia, TRPV1, Axonal sprouting

## Abstract

**Background:**

Electroacupuncture (EA) is effective in relieving pain in patients with postherpetic neuralgia (PHN). However, the mechanism underlying the therapeutic effect of EA in PHN is still unclear. Systemic injection of resiniferatoxin (RTX), an ultrapotent analog of TRPV1 agonist, in adult rats can reproduce the clinical symptoms of PHN by ablating TRPV1-expressing sensory neurons. In this study, we determined the beneficial effect of EA and the potential mechanisms in this rat model of PHN.

**Methods:**

PHN was induced in rats by a single injection of RTX. Thermal hyperalgesia was tested with a radiant heat stimulus, and mechanical allodynia was quantified with von Frey filaments. TRPV1 receptors were shown by using immunofluorescence labeling. The ultrastructural changes of the sciatic nerve were assessed by electron microscopic examination. The sprouting of myelinated primary afferent terminals into the spinal dorsal horn was mapped by using the transganglionic tracer cholera toxin B-subunit (CTB).

**Results:**

RTX injection diminished thermal sensitivity and gradually induced tactile allodynia within 3 weeks. EA applied to GB30 and GB34 at 2 and 15 Hz, but not 100 Hz, significantly increased the thermal sensitivity 4 weeks after treatment and decreased the tactile allodynia 2 weeks after treatment in RTX-treated rats. EA treatment at 2 and 15 Hz recovered the loss of TRPV1-positive dorsal root ganglion neurons and their central terminals of afferent fibers in the spinal superficial dorsal horn of RTX-treated rats. Moreover, EA significantly reduced the loss of unmyelinated fibers and the damage of the myelinated nerve fibers of RTX-treated rats. Furthermore, EA at 2 and 15 Hz inhibited the sprouting of myelinated primary afferent terminals into the spinal lamina II of RTX-treated rats.

**Conclusions:**

EA treatment improves thermal perception by recovering TRPV1-positive sensory neurons and nerve terminals damaged by RTX. EA Also reduces RTX-induced tactile allodynia by attenuating the damage of myelinated afferent nerves and their abnormal sprouting into the spinal lamina II. Our study provides new information about the mechanisms of the therapeutic actions of EA in the treatment of PHN.

## Background

Postherpetic neuralgia (PHN, Shingles) is a neuropathic pain condition characterized by the presence of profound mechanical allodynia and typically occurs in elderly but otherwise healthy individuals [1]. Although electroacupuncture (EA) is effective in relieving pain in patients with PHN [2,3], little is known about the mechanism of how EA produces antinociceptive effects in PHN.

In patients with PHN, profound tactile allodynia and impaired thermal sensitivity often co-exist in the same affected dermatomes [1,4]. We have shown that depletion of capsaicin-sensitive afferents by systemic treatment with a ultrapotent transient receptor potential vanilloid 1 (TRPV1) agonist, resiniferotoxin (RTX), in adult rats produces long-lasting paradoxical changes in mechanical and thermal sensitivities, which resembles the unique clinical features of patients with PHN [5]. The delayed tactile allodynia is likely attributable to damage of myelinated afferent fibers and their abnormal sprouting in lamina II of the spinal dorsal horn. Although mechanical allodynia and hyperalgesia can be induced by inoculation of herpes simplex virus type-1 or varicella-zoster virus on the hindpaw of mice or rats, the viral infection models often fail to induce thermal impairment and have the disadvantage of developing tissue inflammation, skin lesions, and paralysis by the virus spreading in the central nervous system [6,7]. Therefore, the RTX model has been used as a non-viral PHN model to search for new treatments that effectively control this chronic pain condition [8,9].

In this study, we determined the effect of EA with different frequencies on thermal impairment and tactile allodynia in a rat model of RTX-induced PHN. We also examined the effect of EA on the loss of TRPV1-positive dorsal root ganglion (DRG) neurons and central terminals of afferent fibers in the superficial dorsal horn of RTX-treated rats. We then probed the effect of EA on RTX-induced loss of unmyelinated fibers and damage of the myelinated nerve fibers in sciatic nerve and the sprouting of myelinated afferent fibers into the lamina II of the spinal dorsal horn. We tested the hypothesis that EA attenuates RTX-induced damage to sensory nerves to reverse thermal impairment and tactile allodynia.

## Results

### Effects of EA on RTX-induced thermal hypoalgesia and mechanical allodynia

**Figure 1:**
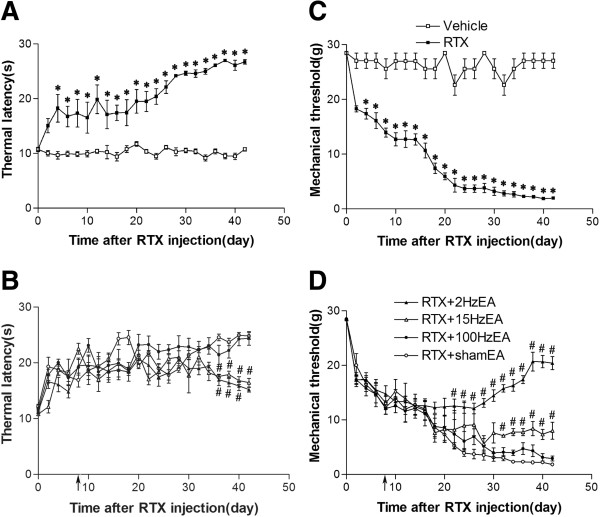
**Time course of the effects of EA on RTX-induced thermal hypoalgesia and mechanical allodynia. A**, Time course of the paw withdrawal latency to a noxious heat stimulus in vehicle- and RTX-treated rats. **B**, Effects of 2, 15, or 100 Hz EA, and sham EA on thermal withdrawal threshold in response to a heat stimulus applied to the left hindpaw of RTX-treated rats. EA was administered for 30 min, once every other day for 5 weeks, starting from 1 week after RTX injection, as indicated by arrows. **C**, Time course of mechanical withdrawal threshold in response to von Frey filaments in vehicle- and RTX-treated rats. **D**, Effects of 2, 15, or 100 Hz EA and sham EA on mechanical withdrawal threshold in response to von Frey filaments applied to the left hindpaw of RTX treated rats. Data are expressed as means ± SEM (n = 10 rats in each group). * P < 0.01, compared with the vehicle group; # P < 0.05, compared with the sham EA group.

The baseline withdrawal thresholds in all the experimental groups before RTX treatment were similar. RTX significantly decreased thermal sensitivity but increased tactile sensitivity 4 days after RTX administration (Figure 1A, C), similar to what we previously reported [5]. EA was applied to GB30 and GB34 for 30 min, once every other day for 5 weeks, starting from 1 week after RTX injection. EA at 2 and 15 Hz, but not 100 Hz, significantly increased the thermal sensitivity 4 weeks after starting EA treatment (Figure 1B).

EA at 2 Hz significantly increased the tactile threshold 2 weeks after starting EA treatment. This effect was enhanced gradually till 5 weeks after starting EA treatment. At 15 Hz, EA also increased the tactile threshold 3 weeks after starting EA treatment compared with that in the sham EA group (Figure 1D). However, no significant changes in the paw withdrawal thresholds were observed in the 100 Hz group during the 6-week period of EA experiment.

### Effect of EA on RTX-induced deletion of TRPV1-expressing DRG neurons and afferent terminals in spinal dorsal horn

**Figure 2:**
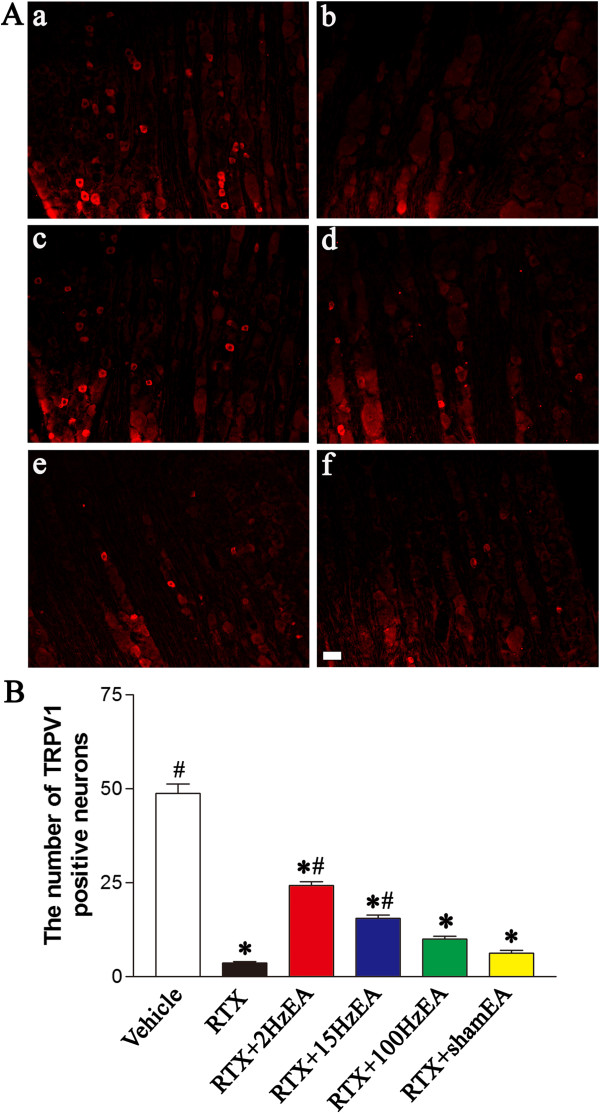
**Effect of EA on RTX-induced deletion of TRPV1-immunoreactive neurons in the DRG. A**, Representative images showing TRPV1-immunoreactive neurons in the lumbar DRG of vehicle (**a**), RTX (**b**), RTX plus 2 Hz EA (**c**), RTX plus 15 Hz EA (**d**), RTX plus 100 Hz EA (**e**), and RTX plus sham EA (**f**) groups. Scale bar, 50 μm. **B**, Summary data show the number of TRPV1 immunoreactive neurons in different groups. Data are expressed as means ± SEM (n = 6 rats in each group). *P < 0.05, compared with the vehicle group; # P < 0.05, compared with the sham EA group.

To examine the effect of EA on TRPV1-expressing primary afferent neurons, TRPV1-immunofluorescent labeling of primary afferent neurons was conducted in the DRG from different groups 5 weeks after EA treatment. TRPV1 immunoreactivity was substantially depleted in small- and medium-sized DRG neurons from RTX-treated rats (Figure 2A). EA with 2 and 15 Hz, but not 100 Hz, recovered the loss of TRPV1-positive DRG neurons induced by RTX injection (Figure 2A, B). The number of TRPV1-positive DRG neurons was significantly higher in 2 and 15 Hz EA groups than in sham EA group (Figure 2B).

**Figure 3:**
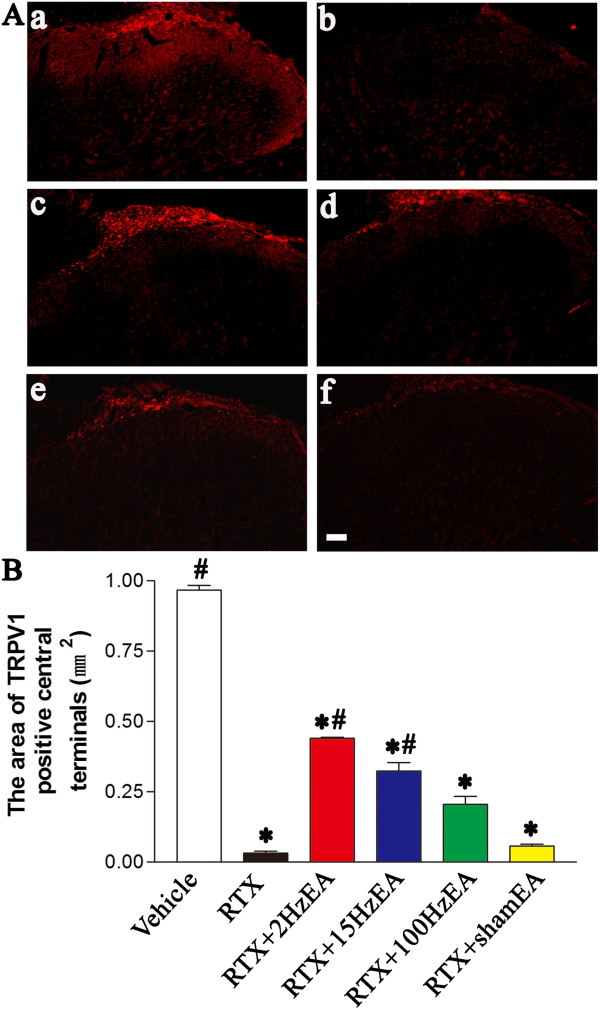
**Effect of EA on RTX-induced deletion of TRPV1 immunoreactive central terminals in the spinal dorsal horn. A**, Representative images showing TRPV1 immunoreactive central terminals of afferent fibers in the spinal dorsal horn of vehicle (**a**), RTX (**b**), RTX plus 2 Hz EA (**c**), RTX plus 15 Hz EA (**d**), RTX plus 100 Hz EA (**e**), and RTX plus sham EA (**f**) groups. Scale bar, 50 μm. **B**, Summary data show the area of TRPV1 immunoreactive central terminals in different groups. Data are expressed as means ± SEM (n = 6 rats in each group). *P < 0.05, compared with the vehicle group; # P < 0.05, compared with the sham EA group.

To determine the effect of EA on TRPV1-expressing central terminals of primary afferents, we also examined TRPV1 immunoreactivity in the spinal dorsal horn. TRPV1 immunoreactivity was completely abolished in dorsal root entry zone and the superficial dorsal horn (in laminae I and II) in RTX-treated rats (Figure 3A, B). EA at 2 and 15 Hz, but not 100 Hz, significantly attenuated the loss of TRPV1-positive central terminals of afferent fibers in the superficial dorsal horn induced by RTX injection (Figure 3A). The area of TRPV1-positive central terminals was significantly larger in 2 and 15 Hz EA groups than in sham EA group (Figure 3B).

### Effect of EA on RTX-induced ultrastructural damage of nerve fibers in the sciatic nerve

**Figure 4:**
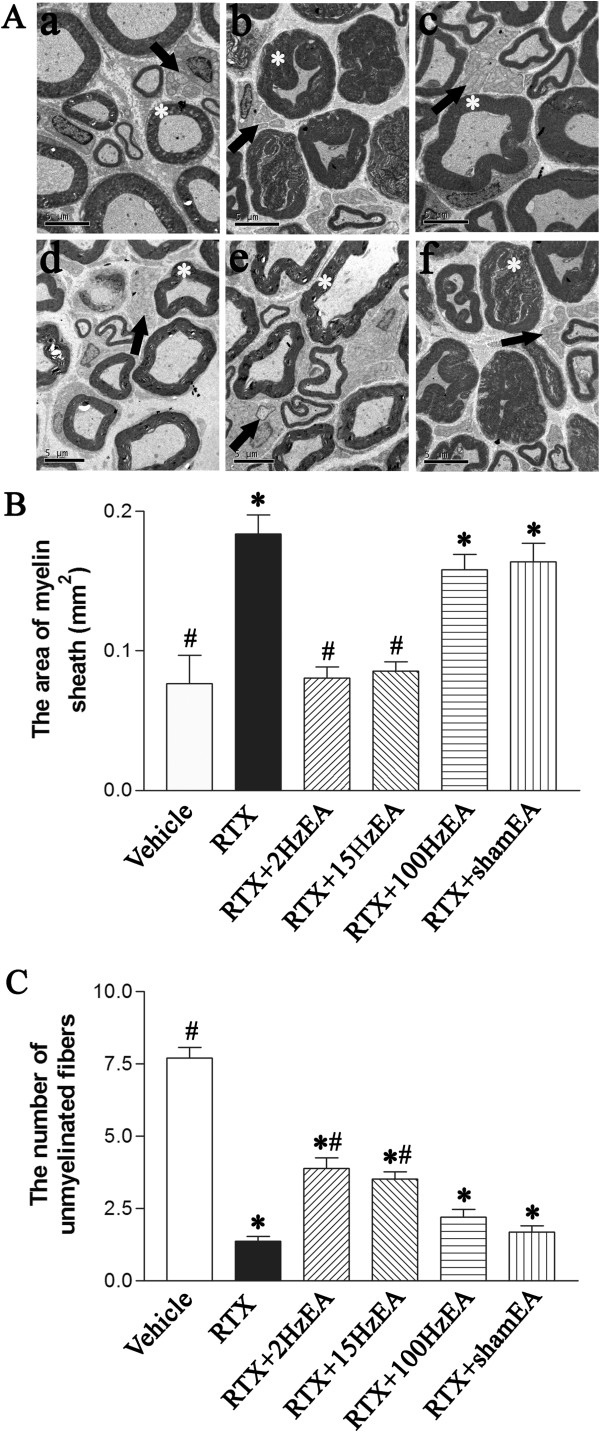
**Effects of EA on RTX-induced ultrastructural changes of myelinated fibers and the number of unmyelinated fibers in the sciatic nerve. A**, Representative electron photomicrographs show ultrastructural changes of myelinated and unmyelinated fibers in the sciatic nerve of vehicle (**a**), RTX (**b**), RTX plus 2 Hz EA (**c**), RTX plus 15 Hz EA (**d**), RTX plus 100 Hz EA (**e**), and RTX plus sham EA (**f**) groups. Scale bar, 5 μm. Asterisk, myelinated fibers; arrow, unmyelinated fibers. **B**, Summary data show the area of myelin sheath of myelinated fibers in the sciatic nerve in different groups. **C**, Summary data show the number of unmyelinated fibers in the sciatic nerve section in different groups. Data are expressed as means ± SEM (n =3 rats in each group). *P < 0.05, compared with the vehicle group; # P < 0.05, compared with the sham EA group.

Electron microscopic examination of the sciatic nerve sections revealed the characteristic appearance of myelinated and unmyelinated fibers. As shown in Figure 4A, RTX induced substantial depletion of unmyelinated fibers and evident damage to myelinated fibers in the sciatic nerve, including axonal swelling, altered myelination, and the appearance of loose and breakdown of the myelin sheath. Compared with the distorted and elongated or wedged-shaped configuration of myelinated fibers in RTX group, the damaged myelinated fibers appeared less swollen and convoluted in shape, and were bounded by a more compact wall of the myelin sheath in EA group. The unmyelinated fibers were recovered after 5 weeks of EA treatment, more unmyelinated fibers can be found in 2 Hz and 100 Hz EA group as arrow indicated (Figure 4A). The area of myelin sheath of myelinated axons per high-power field (800 μm^2^) was significantly increased in RTX-treated rats. EA at 2 and 15 Hz, but not 100 Hz, significantly reduced the area of myelin sheath, compared with the sham EA group (Figure 4B). Also, EA at 2 and 15 Hz significantly increased the number of unmyelinated axons per high-power fields (800 μm^2^) compared with that in the sham EA group (Figure 4C).

### Effect of EA on RTX-induced sprouting of myelinated afferent fibers labeled by cholera toxin B subunit (CTB) in the dorsal horn

**Figure 5:**
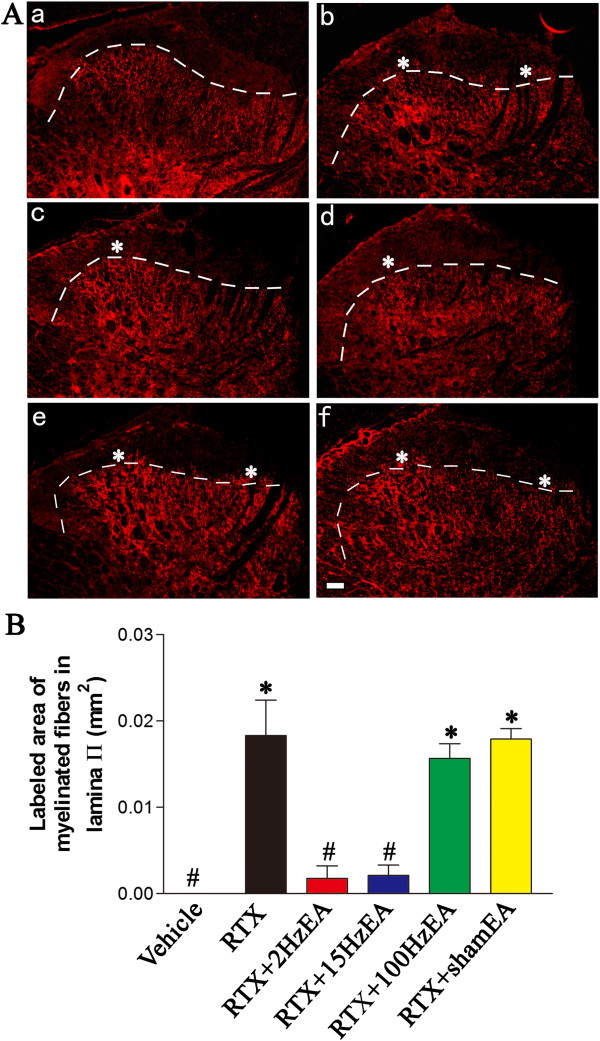
**Effect of EA on RTX-induced sprouting of myelinated afferent fibers in the spinal lamina II. A**, Representative images show CTB immunoreactive myelinated fibers in the spinal dorsal horn of vehicle (**a**), RTX (**b**), RTX plus 2 Hz EA (**c**), RTX plus 15 Hz EA (**d**), RTX plus 100 Hz EA (**e**), and RTX plus sham EA (**f**) groups. Right side is the medial side of dorsal horn in all the images. Scale bar, 50 μm. Dotted line marks the division separating lamina II and III. Asterisk, sprouting of myelinated fibers in lamina II. **B**, Summary data show the area of CTB-labeled afferent terminals in lamina II in different groups. Data are expressed as means ± SEM (n = 3 rats in each group). *P < 0.05, compared with the vehicle group; # P < 0.05, compared with the sham EA group.

Preferential labeling of myelinated fibers by transganglionic transporting CTB allowed us to determine whether EA had any effect on the sprouting of myelinated afferent fibers in the spinal dorsal horn induced by RTX. In vehicle-treated group, CTB labeling was quite dense in the medial part of laminae III-V, but was absent in the laminae I-II (Figure 5A). Some CTB-labeled myelinated afferent fibers sprouted into lamina II in RTX-treated rats, similar to what we have shown before [5]. After RTX treatment, CTB-labeled nerve terminals were more intense in the medial part of lamina II, and relatively sparse in the lateral part of lamina II. The area of myelinated fibers labeled by CTB in lamina II of RTX-treated rats was significantly increased than that in the vehicle control group.

EA at 2 and 15 Hz significantly attenuated the extension of CTB-labeled terminals into lamina II. Compared with the sham EA group, spinal cord sections from 2 and 15 Hz EA-treated rats had almost no CTB labeling in the medial part of lamina II but still contained scattered CTB labeling in the lateral part of lamina II. However, the RTX-induced sprouting of myelinated afferent fibers into lamina II remained evident and unchanged in rats treated with 100 Hz EA (Figure 5A). EA at 2 and 15 Hz significantly decreased the area of CTB-labeled myelinated fibers in lamina II compared with that in the sham EA group (Figure 5B).

## Discussion

In the present study, we demonstrate that one single injection of RTX substantially reduced thermal sensitivity but produced profound and persistent tactile allodynia in adult rats. These symptoms are similar to those seen in patients with PHN [1,4]. EA at 2 and 15 Hz, but not 100 Hz, significantly recovered the thermal sensitivity of RTX-treated rats 4 weeks after EA treatment. EA at 2 and 15 Hz also significantly decreased the tactile allodynia of RTX-treated rats after 2 weeks of EA treatment. Consistently, EA with 2 and 15 Hz attenuated the loss of TRPV1-positive DRG neurons and the central terminals of afferent fibers in the superficial dorsal horn of RTX-treated rats. EA at 2 and 15 Hz also significantly attenuated the loss of unmyelinated fibers and the damage of the myelinated nerve fibers of RTX-treated rats. Furthermore, EA with 2 and 15 Hz, but not 100 Hz, inhibited the sprouting of myelinated afferent terminals into lamina II of the spinal dorsal horn of RTX-treated rats. Thus, our study suggests that EA improves thermal and mechanical sensitivities in a rat model of PHN by attenuating RTX-induced damage to sensory nerves and associated anatomical plasticity in the peripheral nerve and spinal dorsal horn.

TRPV1 plays an important role in detecting thermal nociception [10]. Resiniferotoxin (RTX), originally isolated from the cactus-like plant Euphorbia resinifera, is an ultrapotent TRPV1 agonist [11]. Systemic injection of RTX ablates TRPV1-expressing sensory neurons and induces a long-lasting impairment of thermal nociception in adult rats [5,12]. In this study, EA treatment had no significant effect on thermal impairment in RTX-treated rats until 4 weeks after EA treatment. The thermal withdrawal threshold of the hindpaw tested was significantly increased within 2 days after RTX administration, whereas EA was applied only 1 week after RTX treatment when most of TRPV1-positive afferent neurons had been depleted by RTX [5]. EA may not rescue TRPV1-positive DRG neurons already damaged by RTX, which may explain no obvious effect of EA until 4 weeks after treatment. However, at the end of 4 weeks of EA treatment, 2 and 15 Hz EA alleviated the reduction of TRPV1-positive primary sensory neurons and their central terminals in the superficial dorsal horn. Moreover, 2 and 15 Hz EA recovered the loss of unmyelinated fibers caused by RTX. Therefore, EA may improve thermal sensitivity by promoting the regeneration and recovery of TRPV1-positive sensory neurons and their central terminals.

EA at 2 and 15 Hz had a more pronounced effect than 100 Hz EA on recovering TRPV1-expressing unmyelinated afferent terminals damaged by RTX. EA can modulate TRPV1 receptors and thermal sensitivity [13-15]. Previous studies have shown that 2 Hz EA can inhibit the streptozotocin- or NGF-induced thermal hyperalgesia and increase TRPV1 expressions in the spinal cord or hindpaw skin [13,14]. Also, EA can promote nerve regeneration after the transection of the sciatic nerve and function of denervated muscle tissues after sciatic nerve lesion [8]. Thus, low frequency EA may be more effective than high frequency EA on regeneration of physically or chemically injured sensory neurons and nerves. EA also could promote restoration of sensory function following partial DRG ganglionectomies by enhancing nerve regeneration from the spared DRG [16,17]. Nevertheless, the biochemical mechanism underlying the beneficial effect of EA in promoting the recovery of sensory neurons and axonal regeneration remains to be explored [18].

We found that EA at 2 and 15 Hz were more effective than 100 Hz on alleviating RTX-induced tactile allodynia. This is consistent with the report by Hwang et al. [19] showing that 2 Hz EA relieves the sign of mechanical allodynia in a rat model of neuropathic pain, while 100 Hz EA has no effect [20]. To determine the potential sites of the action of EA, we examined the effect of EA on the peripheral nerve and the topographical projection of myelinated afferent terminals in the spinal dorsal horn. Our analyses showed that EA attenuated ultrastructural damage to the myelinated fibers in the sciatic nerve in RTX-treated rats. This finding suggests that the therapeutic effect of EA may not be confined to the unmyelinated C-fibers. Since TRPV1 receptors are also located in 30% of myelinated A-fiber afferent neurons [21], damage to this population of myelinated afferent nerves may play an important role in allodynia development induced by RTX. Previous studies have shown that injured myelinated afferent nerves develop ectopic activities that could alter and amplify the sensory input so that an innocuous stimulus could be interpreted as being painful [22,23]. Thus, these ectopic discharges from damaged myelinated afferents could induce and maintain a state of hypersensitivity of spinal dorsal horn neurons, thereby resulting in allodynia. In our study, 2 and 15 Hz EA significantly reduced the tactile allodynia in RTX-treated rats, which could be explained, at least in part, by attenuating the damage to myelinated afferent fibers by RTX.

Another salient finding of our present study is that 2 and 15 Hz, but not 100 Hz, EA inhibited sprouting of CTB-labeled terminals of myelinated afferent terminals in the spinal lamina II in RTX-treated rats, which is parallel to the inhibitory effect of EA on tactile allodynia. To our knowledge, this is the first study showing that the association of the EA effects on tactile allodynia and sprouting of myelinated afferent fibers in a PHN model. The relationship between sprouting of myelinated fibers and allodynia has been well demonstrated previously. Pain hypersensitivity after chronic constriction nerve injury is associated with the sprouting of myelinated fibers into the lamina II [24]. Moreover, it has been demonstrated that excessive sprouting of myelinated fibers within the dorsal horn after spinal cord injury was associated with neuropathic pain [25]. In our study, using CTB as a tracer for myelinated afferent fibers, we confirmed that myelinated fibers sprouted into lamina II after RTX treatment. Lamina II normally receives mostly nociceptive C-fiber afferent inputs, and the expansion of myelinated afferent fibers into lamina II could alter the peripheral input to produce a painful response to touch [26,27]. Because 2 and 15 Hz EA inhibited the sprouting of myelinated nerve terminals 5 weeks after EA treatment, EA may reduce tactile allodynia through inhibition of primary afferent nerve sprouting in the spinal lamina II [28]. However, the mechanism through which EA regulates myelinated fiber sprouting remains to be defined. In RTX-treated rats, TRPV1-expressing unmyelinated afferent nerve terminals in the lamina II are largely removed, and the presence of vacant synaptic sites within the superficial dorsal horn may promote sprouting from neighboring intact myelinated terminals [26,27]. Because EA treatment recovered TRPV1-expressing unmyelinated afferent terminals in RTX-treated rats, EA could decrease vacant synaptic sites and thus minimize axonal sprouting of myelinated afferent fibers. Therefore, EA-produced reorganization of the spinal dorsal horn circuitry may constitute a neuroanatomic basis for the potent therapeutic effect of EA on nociceptive processing in PHN.

## Conclusions

In summary, we found that 2 and 15 Hz EA improved thermal sensitivity by promoting regeneration of TRPV1-positive unmyelinated sensory neurons damaged by RTX. Moreover, 2 and 15 Hz EA reduced RTX-induced tactile allodynia, an effect that is probably attributable to attenuating the damage to TRPV1-expressing myelinated afferent nerves and inhibiting their abnormal sprouting into lamina II of the spinal dorsal horn. These new findings greatly improve our current knowledge of the mechanisms underlying the therapeutic effect of EA in PHN.

## Methods

### Animal models

Experiments were carried out on male adult Sprague–Dawley rats (250–280 g) purchased from Experimental Animal Center of Tongji Medical College of Huazhong University of Science and Technology. All procedures were approved by the Animal Care Committee at Huazhong University of Science and Technology and conformed to the ethical guidelines of the International Association for the Study of Pain [29]. The rats were individually housed in cages with a 12-hr light/dark cycle and had free access to food and water. Each rat in the RTX group received a single intraperitoneal injection of RTX (250 μg/kg, LC Laboratories, Woburn, MA) under halothane (2% in O_2_) anesthesia. RTX was dissolved in a mixture of 10% Tween 80 and 10% ethanol in normal saline [30]. Separate rats received a mixture of 10% Tween 80 and 10% ethanol in normal saline and were used as the vehicle control. Before RTX or vehicle treatment, the baseline sensitivity of each rat to mechanical and thermal stimulation was measured.

### Electroacupuncture (EA) treatment

In the EA treatment group, the rats received EA administration on the left “Huantiao” (GB30) and “Yanglingquan” (GB34) once every other day, starting from 1 week after RTX injection for 5 weeks. EA (1 mA and 0.1 ms) was administered at different frequency (2 Hz, 15 Hz or 100 Hz) for 30 min. Current was delivered with a modified current-constant Han’s Acupoint Nerve Stimulator (LH202, Huawei Co.Ltd., Beijing, China). GB30 and GB34 were chosen based on their effective use in reducing inflammatory pain in rats [31,32].

Two acupuncture needles were inserted into two acupoints corresponding to GB30 and GB34 in humans. GB30 is located at the junction of the lateral 1/3 and medial 2/3 of the distance between the greater trochanter and the hiatus of the sacrum; and GB34 lies on the lateral aspect of the leg, in the depression anterior and inferior to the head of the fibula in rats [33]. During EA treatment, each rat was placed in an inverted clear plastic chamber (approximately 4 × 4 × 11 cm^3^) but was not restrained. The animals remained still during EA treatment and showed no evident signs of distress. For sham control, acupuncture needles were inserted ipsilaterally into GB30 and GB34 for 30 min without electrical stimulation or manual needle manipulation.

### Nociceptive behavioral tests

The behavioral tests were performed 3 times before RTX injection and once every other day, starting from 1 week after RTX injection. The animals were habituated to the testing environment for 30 min. Thermal sensitivity was assessed by exposing the mid-plantar surface of the hindpaw to a beam of radiant heat through a transparent glass surface using a plantar analgesia meter (UgoBasile, Italy), as previously described [34]. The mean value of the withdrawal latency on two to three consecutive trials was calculated. A cutoff of 30 sec was used to avoid potential tissue damage [30].

Mechanical allodynia was assessed by placing rats on an elevated mesh floor, and the tactile threshold was measured by using the “up-down” method (Chaplan et al., 1994; Chen and Pan, 2002). After an acclimation period of 30 min, a series of calibrated von Frey filaments (Stoelting, Wood Dale, IL) were applied perpendicularly to the plantar surface of both hindpaws with sufficient force to bend the filament for 6 sec. Brisk withdrawal or paw flinching was considered as a positive response. The test was repeated two to three times in each rat, and the mean value was calculated.

### Immunofluorescence labeling of TRPV1 receptors

Immunofluorescence labeling of TRPV1 receptors in left DRG (ipsilateral to EA treatment) and the spinal cord of vehicle-, RTX-, EA-, and sham EA-treated rats were performed to determine the effect of EA on RTX-induced deletion of TRPV1 receptors. After 5 weeks of EA treatment (6 weeks after RTX injection), six rats in each group were deeply anesthetized with 10% chloralic hydras (3.5 ml/kg, i.p.) and transcardially perfused with 500 ml 37°C normal saline followed by 500 ml 4°C paraformaldehyde in 0.1 M phosphate buffer (pH 7.4). The lumbar L4-L6 segment of the spinal cord and DRG were quickly removed and postfixed for 2 hr in the same fixative solution and cryoprotected in 30% sucrose in 0.1 M phosphate buffer for 48 hr at 4°C. The sections were cut at 25 μm on a cryostat, which were mounted onto gelatin-coated slides and air dried overnight. The sections were rinsed in 0.1 M PBS and blocked for 1 hr with 5% donkey serum and 0.2% tween-20 in PBS, followed by incubation at 37°C for 1 hr then at 4°C overnight with the primary antibody (rabbit anti-TRPV1 N terminal, dilution 1:1000; Neuromics, Minneapolis, MN) diluted in PBS containing 5% donkey serum, 0.3% Triton X-100. Subsequently, sections were washed 4 times with 0.05% Tween-20 in PBS for 5 min, and incubated with a secondary antibody: donkey anti-rabbit IgG conjugated with Dynight 594 (1:500; Jackson ImmunoResearch). Sections were washed 4 times with 0.05% Tween-20 in PBS for 5 min, and then coverslipped. Digital images were acquired using an Olympus BX51 fluorescence microscope (Olympus, Tokyo, Japan). Images were captured using a Qimaging Micropublisher RTV 5.0 microscope camera and QCapture Pro 6.0 software (Qimaging, TX, USA). A total of 5–6 sections from DRG and the spinal cord were randomly selected in each rat. The number of unmyelinated axons in each view field was counted by an investigator in a blind fashion. The area of TRPV1-positive central terminals in each view field was measured by using Image J software (NIH, Bethesda, USA).

### Electron microscopic examination of the sciatic nerve

Electron microscopy was used to assess the ultrastructural changes of myelinated and unmyelinated fibers in left sciatic-nerve sections of vehicle-, RTX-, EA-, and sham EA-treated rats. After 5 weeks of EA treatment, three rats in each groups were anesthetized with 10% chloralic hydras (3.5 ml/kg,i.p), and the left sciatic nerve was surgically removed and fixed in 2.5% glutaraldehyde (Sigma-Aldrich) solution. After 2 hr, tissues were transferred into sodium phosphate buffer solution. The tissue was then postfixed in 1% osmium tetroxide for 2 hr, dehydrated in a series of graded ethanol solutions and dipped in mixtures of acetone and epoxy resin for 2 hr and then infiltrated with pure epoxy resin for 2 hr. Finally, the sections were embedded and polymerized for 10 hr in 80°C thermostats box, and ultrathin sections were cut using a Leica ultramicrotome (Leica Mycrosystems, Heidelberg, Germany), poststained with uranyl acetate and Reynold’ lead citrate, and mounted on mesh grids. Photomicrographs were taken on a transmission electron microscope (Tecnai 12-G2; FEI Company, Eindhoven, Netherlands). To quantify the area of myelinated axons and the number of unmyelinated axons in the sciatic nerve, fifteen high-power fields (800 μm^2^) were randomly selected from each sciatic-nerve section from three rats in each group. The area of myelinated axons in each high-power field was measured by using Image J software. The number of unmyelinated axons in each high-power field was counted by an investigator in a blind fashion.

### Tracing of myelinated afferent fiber projections to the dorsal horn

The transganglionic tracer, cholera toxin B-subunit (CTB) has been used to map the central projections of cutaneous myelinated primary afferents, in the spinal dorsal horn of rats [35]. After 5 weeks EA treatment, CTB (1%, List Biological Laboratories, Campbell, CA) was injected into left sciatic nerve of vehicle-, RTX-, EA- and sham EA-treated rats to determine the effect of EA on RTX-induced sprouting of myelinated afferent fibers labeled by CTB in the dorsal horn. The sciatic nerve was exposed at the midthigh level after the rats were anesthetized with 10% chloralic hydras (3.5 ml/kg, i.p). 4 μl volumes of CTB tracers was loaded into Hamilton microsyringes and injected into the sciatic nerves on left side of the body.

After allowing 4 days for transganglionic axoplasmic transport of the tracer, each rat was deeply anesthetized with 10% chloralic hydras and transcardially perfused with 300–400 ml 37°C normal saline followed by 500 ml 4°C paraformaldehyde in 0.1 M phosphate buffer (pH 7.4). The lumbar L4-L6 segment of the spinal cord was quickly removed and postfixed for 2 hr in the same fixative solution and cryoprotected in 30% sucrose in 0.1 M phosphate buffer for 48 hr at 4°C. The sections were cut (20 μm in thickness) on a cryostat, mounted onto gelatin-coated slides, and air dried. For goat anti-CTB immunofluorescence labeling, the sections were rinsed in 0.1 M PBS and blocked for 1 hr with 5% donkey serum and 0.2% tween-20 in PBS, followed by incubation at 37°C for 1 hr then at 4°C overnight with the primary antibody (goat anti-CTB, dilution 1:800; List Biological Laboratories, Campbell, CA) diluted in PBS containing 5% donkey serum, 0.3% Triton X-100. Subsequently, sections were washed 4 times with 0.05% Tween-20 in PBS for 5 min, and incubated with a secondary antibody: donkey anti-goat IgG conjugated with Dynight 594 (1:500; Jackson ImmunoResearch). Sections were washed 4 times with 0.05% Tween-20 in PBS for 5 min, and then coverslipped. An Olympus BX51 fluorescence microscope was used to view the sections, and images were captured using a Qimaging Camera and QCapture software as described before. A total of 5–6 sections from the spinal cord were randomly selected in each rat. Anatomic outlines of the gray matter of dorsal horn were first plotted and the area of CTB-labeled myelinated fibers in lamina II was measured by using Image J software.

### Statistical analysis

Data are presented as means ± SEM. We used one-way analysis of variance (ANOVA) (the number and area of labeled neurons and nerve fibers) or two-way ANOVA (behavioral data) to determine the overall effect of interventions. Post hoc test (Tukey’s or Bonferroni’s) was then used to determine the statistical difference between individual groups. A P value of less than 0.05 was considered statistically significant.

## Abbreviations

PHN: Postherpetic neuralgia; EA: Electroacupuncture; RTX: Resiniferatoxin; TRPV1: Transient receptor potential vanilloid type1; CTB: Cholera toxin β subunit; DRG: Dorsal root ganglion

## Competing interests

The authors declare that they have no competing interests.

## Authors’ contributions

CW carried out the animal model preparation, nociceptive behavioral tests, tracing of myelinated fiber. ZL conducted nociceptive behavioral tests. YZ and YG helped to perform immunofluorescence labeling procedures. JL and FG conducted EA treatment. XM and BT participated in imaging acquisition and analysis. JS helped to analyze data. HP participated in the study design and manuscript writing. ML conceived of the study, oversaw the design, coordinated the study, and edited the manuscript. All authors read and approved the final manuscript.
